# Serum Calcium as a Determinant of Fibroblast Growth Factor 23 Levels in Patients With Primary Hyperparathyroidism and Graves’ Disease

**DOI:** 10.7759/cureus.93972

**Published:** 2025-10-06

**Authors:** Hiroyuki Yamashita, Yusuke Mori, Yasuo Imanishi, Hisakazu Shindo, Daisuke Tatsushima, Seigo Tachibana, Takashi Fukuda, Hiroshi Takahashi, Yuji Nagayama, Shinya Satoh

**Affiliations:** 1 Surgery, Yamashita Thyroid Hospital, Fukuoka, JPN; 2 Nephrology, Osaka Metropolitan University Graduate School of Medicine, Osaka, JPN; 3 Endocrinology, Yamashita Thyroid Hospital, Fukuoka, JPN

**Keywords:** 1'25-dihydroxyvitamin d, fibroblast growth factor 23 (fgf23), graves’ disease, hypercalcemia, parathyroid hormone, primary hyperparathyroidism

## Abstract

Objective: Fibroblast growth factor 23 (FGF23) is a phosphate-regulating hormone with an emerging role in calcium homeostasis. However, its role in hypercalcemic conditions remains unclear. This study aimed to investigate the relationship between serum calcium and FGF23 levels in two hypercalcemic disorders: primary hyperparathyroidism (PHPT) and Graves’ disease.

Methods: A cross-sectional analysis was performed in 98 patients with PHPT and 45 patients with Graves’ disease. Clinical and biochemical parameters, including parathyroid hormone (PTH), phosphate, and 1,25-dihydroxyvitamin D3 (1,25(OH)_2_D), were analyzed.

Results: In PHPT, serum calcium was the only independent determinant of FGF23 levels (P < 0.0001), whereas in Graves’ disease, both serum calcium (P < 0.001) and 1,25(OH)_2_D (P < 0.005) were independently associated with FGF23. A significant positive correlation between calcium and FGF23 was observed in both conditions. Interestingly, FGF23 levels were elevated despite different phosphate profiles: hypophosphatemia in PHPT and hyperphosphatemia in Graves’ disease.

Conclusions: These findings suggest that hypercalcemia may directly contribute to FGF23 elevation, independent of phosphate levels. However, the compensatory effect of FGF23 in modulating calcium homeostasis appears to be limited. This study provides new insights into the calcium sensitivity of FGF23 in endocrine disorders characterized by altered mineral metabolism.

## Introduction

Fibroblast growth factor 23 (FGF23) is a hormone primarily secreted by osteocytes and osteoblasts that regulates phosphate metabolism [1]. It reduces renal phosphate reabsorption by suppressing sodium-phosphate cotransporters and inhibiting 1,25-dihydroxyvitamin D3 (1,25(OH)_2_D) synthesis, thereby reducing intestinal phosphate absorption [2]. Excess FGF23 activity contributes to hypophosphatemic disorders such as X-linked hypophosphatemia and tumor-induced osteomalacia, whereas FGF23 deficiency or resistance can lead to hyperphosphatemia and ectopic calcifications [3,4].

In addition to phosphate regulation, recent studies have suggested that FGF23 may affect calcium homeostasis. FGF23 can suppress parathyroid hormone (PTH) secretion via fibroblast growth factor receptor 1 (FGFR1) and its co-receptor Klotho. Additionally, it downregulates 1α-hydroxylase while stimulating 24-hydroxylase, leading to decreased 1,25(OH)_2_D levels and reduced calcium absorption. In turn, 1,25(OH)_2_D stimulates FGF23 expression in osteocytes, forming an additional feedback mechanism that integrates phosphate and calcium regulation [5,6]. Animal studies have shown increased FGF23 levels following high-calcium diets or intravenous calcium loading in rodents, indicating that calcium may directly stimulate FGF23 production [7,8]. These findings suggest that FGF23 may play a regulatory role in calcium balance, particularly under hypercalcemic conditions.

Primary hyperparathyroidism (PHPT) and Graves’ disease are both associated with hypercalcemia, although the underlying mechanisms are different. In PHPT, hypercalcemia results from autonomous PTH secretion, whereas in Graves’ disease, it is primarily due to thyroid hormone-induced bone resorption [9,10]. Previous studies focused on the role of FGF23 in phosphate metabolism of patients with PHPT and Graves' disease [11,12]. These studies demonstrated elevated FGF23 levels in patients with hyperthyroidism and inconsistent FGF23 involvement in phosphate regulation in PHPT, suggesting that FGF23 regulation may be context-dependent [11,12].

Given the known suppressive effects of FGF23 on PTH and 1,25(OH)_2_D, we hypothesized that hypercalcemia itself may serve as a stimulus for FGF23 secretion. To explore this, we performed a cross-sectional study of patients with PHPT and Graves’ disease to assess the relationship between serum calcium and FGF23 levels. This study aimed to clarify whether FGF23 functions as a calcium-sensitive hormone in vivo and to assess its potential role in calcium regulation across different hypercalcemic states.

## Materials and methods

Study design and ethical approval

This study was conducted in accordance with the Declaration of Helsinki and approved by the Ethics Committee of Yamashita Thyroid Hospital (approval number: 2025-2). Written informed consent was obtained from all participants in previous studies. As this investigation involved a re-analysis of anonymized samples based on a new hypothesis, no additional consent was required.

Patients

Two cohorts were analyzed. The first included 98 patients with PHPT who underwent parathyroidectomy at Noguchi Thyroid Hospital Foundation between January 2000 and June 2002 (Table 1). Of these, 83 were females, including 65 postmenopausal women. None were receiving estrogen therapy. Clinical manifestations included nephrolithiasis (n = 28), bone fractures (n = 8), and severe fatigue (n = 3). 64 patients (65%) were classified as having asymptomatic PHPT. The mean serum calcium concentration was 11.3 ± 0.7 mg/dL, and the mean weight of resected parathyroid glands was 954 ± 1139 mg. The second cohort comprised 45 patients randomly selected from a group of 568 individuals diagnosed with Graves’ disease in 2002. Patients were selected to represent hyperphosphatemic, hypophosphatemic, and normophosphatemic subgroups and had available frozen serum samples from their initial clinic visit. Of these 45 patients, 27 had no prior antithyroid treatment. Of the 18 previously treated patients, six had received medication for less than one month, six for less than one year, and five for more than one year.

**Table 1:** Clinical and biochemical data from patients with PHPT and Graves’ disease Values are expressed as mean ± SD for normally distributed data and median (IQR) for non-normally distributed data. *: The calcium (mg/dL) level was calculated using the following formula: calcium concentration (mg/dL) + 0.8 (4-albumin (g/dL). PHPT: Primary hyperparathyroidism; T3: Triiodothyronine; T4: Thyroxine; TSH: Thyroid-stimulating hormone; PTH: Parathyroid hormone; FGF23: Fibroblast growth factor 23; ALP: Alkaline phosphatase; eGFR: Estimated glomerular filtration rate; BGP: Bone GLA protein; NTX: Type I collagen cross-linked N-telopeptides; BCE: Bone collagen equivalent; 25(OH)D: 25-hydroxyvitamin D; 1,25(OH)_2_D: 1,25-dihydroxyvitamin D3; ND: Not determined; IQR: Interquartile range

Variables	PHPT patients (n = 98)	Graves’ patients (n = 45)	Reference range
Age	57.2 ± 12.7	44.3 ± 14.2	
Sex (M/F)	15/83	16/29	
Free T3 (pg/mL)	ND	12.2 (8.0–22.5)	2.3–4.3
Free T4 (ng/dL)	ND	3.7 (2.7–7.8)	0.9–1.7
TSH (μU/mL)	ND	0.01 (0.01–0.01)	0.5–5
PTH (pg/mL)	144 (94–225)	31.4 (20.2–47.1)	15–65
FGF23 (ng/L)	32.9 (24.5–40.9)	32.5 (25.9–45.3)	10–50
Calcium (mg/dL)*	11.3 ± 0.7	10.1 ± 0.5	8.8–10.1
Phosphate (mg/dL)	2.97 ± 0.51	4.32 ± 1.28	2.7–4.6
ALP (IU/L)	126 (93–170)	330 (261–447)	38–113
Creatinine (mg/dL)	0.7 (0.6-0.8)	0.6 (0.5–07)	0.46–0.79
eGFR (ml/min/1.73m^2^)	74.6 ± 19.4	64.9 ± 16.4	>60
BGP (ng/mL)	20.5 (17.0–25.3)	ND	2.5–13
Urinary NTX (nmol BCE/mmol creatinine)	112 (76–183)	ND	14–100
25(OH)D (ng/mL)	14.6 ± 5.5	ND	>30 ng/mL
1,25(OH)_2_D (pg/mL)	65.8 (52.0–89.2)	45.1 ± 23.4	20–60
Parathyroid gland weight (mg)	532 (304–1,062)	ND	

Clinical and biochemical assessment

Fasting blood samples were obtained from patients with PHPT the morning after hospital admission and from Graves’ disease patients at their first clinic visit. Serum levels of total calcium, albumin, and phosphate were measured using standard automated methods. Serum alkaline phosphatase (ALP) was measured using the Japan Society of Clinical Chemistry (JSCC) method, which was the standard clinical practice in Japan at the time of data collection (2000). For international comparison, JSCC values can be approximately converted to International Federation of Clinical Chemistry and Laboratory Medicine (IFCC) values by multiplying by 0.645.

Albumin-corrected calcium was calculated as follows:

Corrected calcium (mg/dL) = measured calcium + 0.8 × (4.0 - albumin (mg/dL))

Serum intact PTH was measured by an electrochemiluminescence immunoassay (ECLusys 2010, Roche Diagnostics, Germany). Serum 25-hydroxyvitamin D (25(OH)D) was determined by a competitive protein binding assay following purification by high-performance liquid chromatography [13]. Serum 1,25(OH)_2_D was measured using a receptor binding assay using bovine mammary gland receptor protein [14]. Serum bone GLA protein (BGP) was measured by immunoradiometric assay (IRMA; Mitsubishi Yuka, Japan). Urinary N-telopeptide of type I collagen (NTX) was measured by ELISA (Osteomark, Ostex International, USA), and values were corrected for urinary creatinine. Serum FGF23 levels were measured using a two-site sandwich ELISA (reference range: 10-50 ng/L), as previously described [15].

Statistical analysis

Statistical analyses were performed with JMP software version 17.0 (SAS Institute Inc., USA). Normally distributed data are presented as mean ± SD, and non-normally distributed data as median with interquartile range (IQR). Data distribution was assessed using the Anderson-Darling test and Q-Q plots. Associations between log-transformed FGF23 or calcium levels and other variables were assessed using univariate linear regression. Variables with P < 0.05 in univariate analysis were included in multivariate linear regression models to identify independent predictors. Spearman’s rank correlation was used to evaluate relationships among calcium-related variables (FGF23, calcium, phosphate, PTH, and 1,25(OH)_2_D). Statistical significance was set at P < 0.05.

## Results

Determinants of FGF23 and serum calcium levels

Univariate and multivariate linear regression analyses identified factors independently associated with serum FGF23 levels (Tables 2, 3).

**Table 2:** Univariate and multivariate analyses of serum variables associated with calcium levels in patients with PHPT and Graves’ disease *: In univariate analysis, simple linear regression was used; **: To avoid confounding between related variables, the multivariate analysis used free T4 as a representative marker of thyroid function and serum creatine as an indicator of renal function; #: Significant at P < 0.05 PHPT: Primary hyperparathyroidism; T3: Triiodothyronine; T4: Thyroxine; TSH: Thyroid-stimulating hormone; PTH: Parathyroid hormone; ALP: Alkaline phosphatase; eGFR: Estimated glomerular filtration rate; BGP: Bone GLA protein; NTX: Type I collagen cross-linked N-telopeptides; 25(OH)D: 25-hydroxyvitamin D; 1,25(OH)_2_D: 1,25-dihydroxyvitamin D3; FGF23: Fibroblast growth factor 23; ND: Not determined

Variables	PHPT patients		Graves’ patients	
	*Univariate	Multivariate	*Univariate	Multivariate
Age	0.6955	-	0.7747	-
Sex	0.4385	-	0.2794	-
Free T3	ND	-	0.0007^#^	-
Free T4	ND	-	< 0.0001^#^	**0.0458^#^
PTH	< 0.0001^#^	0.0005^#^	< 0.0002^#^	0.4086
Phosphate	< 0.0001^#^	0.0751	< 0.0001^#^	0.0342^#^
ALP	0.205	-	0.3918	-
Creatinine	0.0124^#^	**0.5307	0.4587	-
eGFR	0.0210^#^	-	0.9152	-
BGP	0.5964	-	ND	-
Urinary NTX	0.0170^#^	0.0456^#^	ND	-
25(OH)D	0.8387	-	ND	-
1,25(OH)_2_D	0.4357	-	< 0.0001^#^	0.2869
FGF23	< 0.0001^#^	< 0.0001^#^	< 0.0001^#^	< 0.0001^#^

**Table 3:** Univariate and multivariate analysis of serum variables associated with log-transformed FGF23 levels in patients with PHPT and Graves’ disease *: In univariate analysis, simple linear regression was used; **: To avoid confounding between related variables, the multivariate analysis included free T4 as an indicator of thyroid function and eGFR as a marker of renal function; ^#^: Significant at P < 0.05 FGF23: Fibroblast growth factor 23; PHPT: Primary hyperparathyroidism; T3: Triiodothyronine; T4: Thyroxine; TSH: Thyroid-stimulating hormone; PTH: Parathyroid hormone; ALP: Alkaline phosphatase; eGFR: Estimated glomerular filtration rate; BGP: Bone GLA protein; NTX: Type I collagen cross-linked N-telopeptides; 25(OH)D: 25-hydroxyvitamin D; 1,25(OH)_2_D: 1,25-dihydroxyvitamin D3, ND: Not determined

Variables	PHPT patients		Graves’ patients	
	*Univariate	Multivariate	*Univariate	Multivariate
Age	0.203	-	0.2558	-
Sex	0.8711	-	0.4446	-
Free T3	ND	-	0.0047^#^	-
Free T4	ND	-	0.0029^#^	**0.5479
PTH	0.1247	-	0.0009^#^	0.8693
Calcium	< 0.0001^#^	< 0.0001^#^	< 0.0001^#^	0.0006^#^
Phosphate	0.0432	0.6407	0.0003^#^	0.8481
ALP	0.2094	-	0.6902	-
Creatinine	0.0007^#.^	-	0.4969	-
eGFR	0.0007^#^	**0.0712	0.1364	-
BGP	0.0149	0.8287	ND	-
Urinary NTX	0.7508	-	ND	-
25(OH)D	0.4909	-	ND	-
1,25(OH)_2_D	0.0405	0.0749	< 0.0001*	0.0019^#^

In patients with PHPT, serum calcium was the only independent determinant of log-transformed FGF23 (β = 0.45, P < 0.0001). In patients with Graves’ disease, both serum calcium (β = 0.58, P < 0.001) and 1,25(OH)_2_D (β = 0.42, P < 0.005) were independently associated with FGF23 levels.

Regarding the determinants of serum calcium, significant associations were observed with PTH (P < 0.001), FGF23 (P < 0.005), urinary NTX (P < 0.01), and ALP (P < 0.05) in PHPT, and with FGF23 (P < 0.0001) and phosphate (P < 0.05) in Graves’ disease.

Correlation analyses

**Figure 1:**
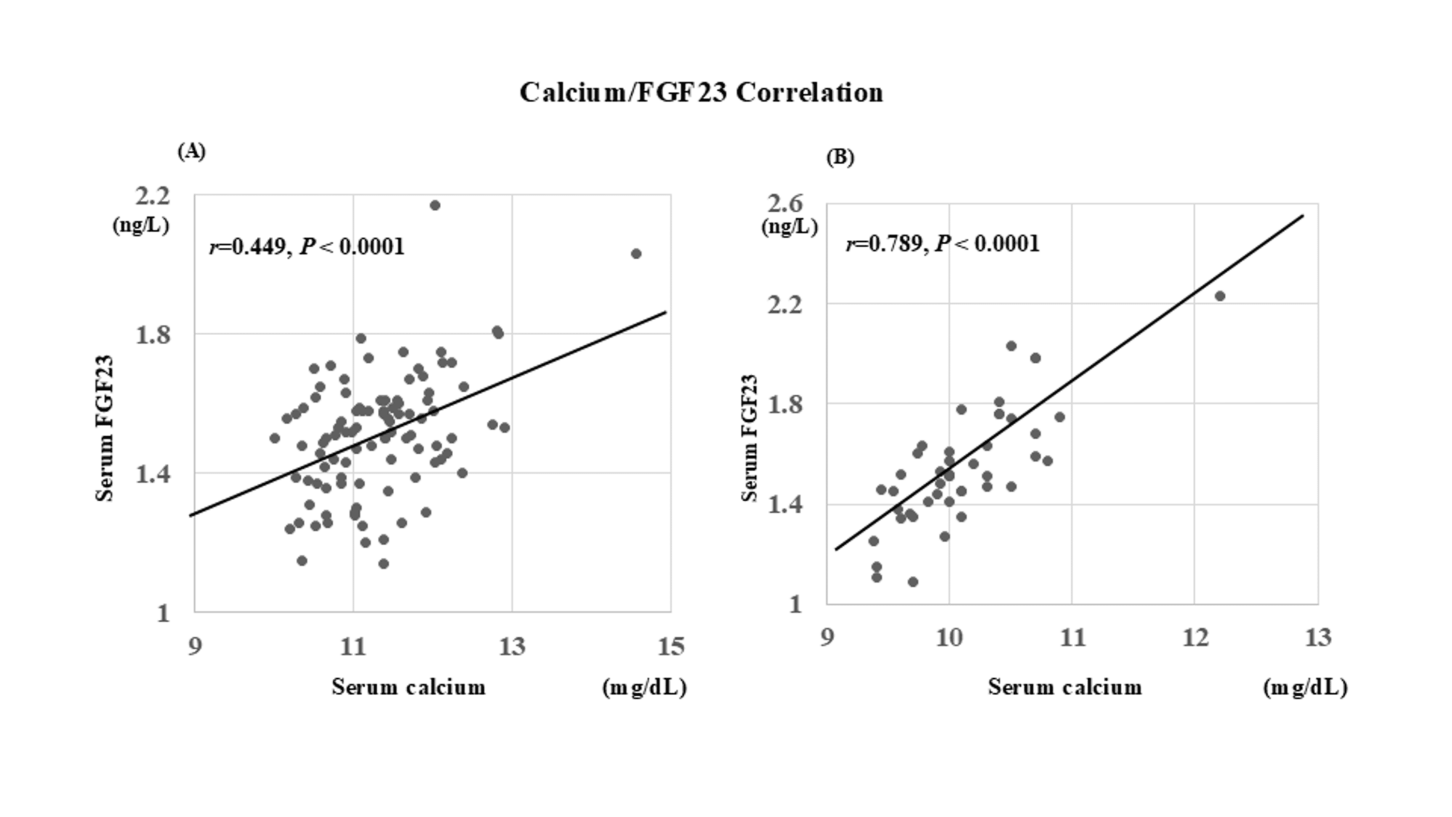
Correlation between serum calcium and log-transformed FGF23 levels in patients with (A) PHPT and (B) Graves’ disease A significant positive correlation was observed in both groups (r = 0.449, P < 0.0001 in PHPT; r = 0.789, P < 0.0001 in Graves’ disease). Axes represent serum calcium (mg/dL) and log-transformed FGF23 (ng/L). FGF23: Fibroblast growth factor 23; PHPT: Primary hyperparathyroidism

A significant positive correlation was observed between log-transformed FGF23 and serum calcium levels in both disease groups (Figure 1).

Spearman correlation matrices for five calcium-related parameters (serum calcium, phosphate, PTH, 1,25(OH)_2_D, and FGF23) are shown in Table 4. In PHPT, 1,25(OH)_2_D was not significantly correlated with either calcium or phosphate, whereas other parameters showed significant correlations in both groups. Despite elevated calcium in both conditions, phosphate levels differed: patients with PHPT had hypophosphatemia, whereas patients with Graves’ disease exhibited hyperphosphatemia.

**Table 4:** Correlations among calcium-related parameters in patients with PHPT Spearman’s rank correlation coefficient was used to assess the relationships among calcium-related factor. PHPT: Primary hyperparathyroidism; PTH: Parathyroid hormone; 1,25(OH)_2_D: 1,25-dihydroxyvitamin D3; FGF23: Fibroblast growth factor 23

	Calcium	Phosphate	PTH	1,25(OH)_2_D	FGF23
Calcium	-	P < 0.0001, r = -0.4115	P < 0.0001, r = 0.5793	P = 0.4467	P < 0.0001, r = 0.4706
						Phosphate	P < 0.0001, r = 0.4115	-	P < 0.0001, r = -0.4644	P = 0.5305	P < 0.0238, r = -0.2282
PTH	P < 0.0001, r = 0.5793	P < 0.0001, r = -0.4644	-	P = 0.0028, r = 0.2989	P = 0.0253, r = 0.2260						
						1,25(OH)_2_D	P = 0.4467	P = 0.5305	P = 0.0280, r = 0.0299	-	P = 0.0247, r = -0.1572
FGF23	P < 0.0001, r = 0.4707	P = 0.0238, r = -0.2282	P = 0.0253, r = 0.2260	P = 0.0247, r = -0.1572	-

Association with hydroxylase activity and PTH efficiency

**Figure 2:**
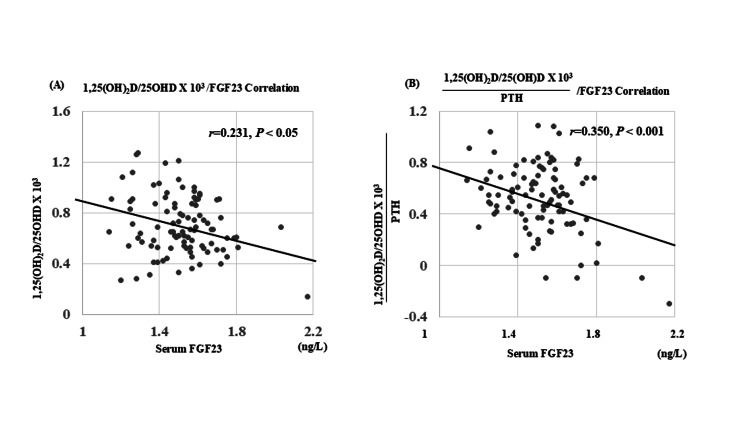
Associations between serum FGF23 and indices of renal hydroxylase activity and PTH efficiency in patients with PHPT (A) Negative correlation between FGF23 and the ratio of 1,25(OH)_2_D to 25(OH)D × 10³, representing composite hydroxylase activity. (B) Negative correlation between FGF23 and the ratio of 1,25(OH)_2_D to 25(OH)D × 10⁵/PTH, indicating the efficiency of PTH-mediated activation of vitamin D. All variables were log-transformed prior to analysis. FGF23: Fibroblast growth factor 23; 1,25(OH)_2_D: 1,25-dihydroxyvitamin D3; 25(OH)D: 25-hydroxyvitamin D; PTH: Parathyroid hormone; PHPT: Primary hyperparathyroidism

Figure 2 illustrates the relationship between serum FGF23 and indices reflecting renal hydroxylase activity and PTH efficiency.

In PHPT, FGF23 levels were inversely correlated with the ratio of 1,25(OH)_2_D to 25(OH)D × 10³ (r = -0.35, P < 0.001), a composite index representing both 1α- and 24-hydroxylase activities (Figure 2A). A similar inverse correlation was observed between FGF23 and the ratio of 1,25(OH)_2_D to 25(OH)D × 10⁵/PTH (r = -0.23, P < 0.05), an index reflecting the efficiency of PTH-mediated vitamin D activation (Figure 2B). These findings suggest a potential regulatory role of FGF23 in attenuating hydroxylase activity and PTH responsiveness under hypercalcemic conditions.

Factors independently associated with log-transformed serum FGF23 and calcium levels are shown in Tables 2 and 3. In PHPT, serum calcium remained the only independent predictor of FGF23 levels (P < 0.0001), whereas in Graves' disease, both serum calcium (P < 0.001) and 1,25(OH)_2_D (P < 0.005) were significant contributors. For serum calcium, key predictors included PTH (P < 0.001), FGF23 (P < 0.005), urinary NTX (P < 0.01), and ALP (P <0.05) in PHPT, and with FGF23 (P < 0.0001) and phosphate (P < 0.05) in patients with Graves' disease. A significant positive correlation between log-transformed FGF23 and serum calcium was consistent across both conditions (Figure 1).

The matrices for Graves’ disease are shown in Table 5.

**Table 5:** Correlations among calcium-related parameters in patients with Graves’ disease Spearman’s rank correlation coefficient was used to assess the relationships among calcium-related factor. PTH: Parathyroid hormone; 1,25(OH)_2_D: 1,25-dihydroxyvitamin D3; FGF23: Fibroblast growth factor 23

	Calcium	Phosphate	PTH	1,25(OH)_2_D	FGF23
Calcium	-	P < 0.0001, r = 0.6331	P = 0.0002, r = -0.5258	P < 0.0001, r = -0.7149	P < 0.0001, r = 0.8059
Phosphate	P < 0.0001, r = 0.6331	-	P = 0.0007, r = 0.4854	P = 0.0001, r = -0.5396	P = 0.0012, r = 0.4688
PTH	P = 0.0002, r = -0.5258	P = 0.0007, r = -0.4854	-	P < 0.0001, r = 0.5647	P = 0.0043, r = -0.3840
						1,25(OH)_2_D	P < 0.0001, r = -0.7149	P = 0.0001, r = -0.5396	P < 0.0001, r = 0.5647	-	P < 0.001, r = -0.6588
-										
FGF23	P < 0.0001, r = 0.80591	P = 0.0012, r = 0.4688	P = 0.0043, r = -0.4182	P < 0.0001, r = -0.6588	-

In PHPT, 1,25(OH)_2_D was not significantly correlated with calcium or phosphate, whereas other parameters showed significant correlations in both conditions (Table 4). During hypercalcemia, serum calcium and FGF23 levels moved in the same direction in both diseases, but the others changed in the opposite direction.

## Discussion

This study identified serum calcium as a significant independent determinant of circulating FGF23 levels in both PHPT and Graves’ disease, supporting the hypothesis that FGF23 may respond to hypercalcemia under certain pathological conditions. Although FGF23 has been traditionally characterized as a phosphaturic hormone, our findings suggest a broader regulatory role, particularly in sustained hypercalcemia. In PHPT, our results are consistent with previous studies by Imanishi et al. and Witteveen et al., who reported positive associations between serum calcium and FGF23 levels [16,17]. In Graves’ disease, we observed an even stronger correlation between calcium and FGF23 than between phosphate and FGF23, which is a novel finding. These results suggest that hypercalcemia rather than phosphate imbalance may be a key driver of FGF23 elevation in both conditions.

Mechanistically, FGF23 may contribute to calcium homeostasis through indirect actions, including PTH secretion inhibition via FGFR1-Klotho signaling, suppression of 1,25(OH)_2_D synthesis, and promotion of 1,25(OH)_2_D catabolism [2,18,19]. These effects could collectively limit intestinal calcium absorption and renal calcium reabsorption. However, in both PHPT and Graves’ disease, hypercalcemia persisted despite elevated FGF23 levels, suggesting that the calcium-lowering effect of FGF23 may be insufficient or overridden. In PHPT, autonomous PTH secretion continues despite high calcium and FGF23 levels, whereas in Graves’ disease, ongoing hyperthyroid bone resorption may maintain elevated calcium and phosphate levels, even with suppressed PTH and 1,25(OH)_2_D levels.

Our previous study showed that FGF23 levels decreased following antithyroid therapy in hyperphosphatemic Graves’ disease, suggesting phosphate-dependent regulation [12]. However, the current results implicate hypercalcemia as a stronger contributor. This discrepancy may reflect concurrent changes in calcium and phosphate during treatment. In PHPT, hypophosphatemia results from PTH-induced phosphaturia, whereas Graves’ disease is often associated with hyperphosphatemia due to increased bone turnover and enhanced renal phosphate reabsorption [9,20]. Despite these opposing phosphate profiles, both groups exhibited elevated FGF23 levels, reinforcing the notion that phosphate imbalance alone does not fully explain FGF23 regulation in these settings.

The inverse correlation between FGF23 and functional indices of renal vitamin D hydroxylase activity, including the ratio of 1,25(OH)_2_D to 25(OH)D × 10⁵/PTH, suggests that FGF23 may attenuate both vitamin D activation and PTH efficiency (Figure 2). Although these indices are not validated biomarkers, they conceptually estimate the interplay between substrate availability and hormonal stimulation. Our findings support a model in which FGF23 contributes to limiting vitamin D-mediated calcium absorption in hypercalcemia.

In our previous study, FGF23 levels remained unchanged after parathyroidectomy in PHPT, despite normalization of serum calcium and PTH levels [11]. This discrepancy may reflect differences in time scales, as measurements after parathyroidectomy may not have captured delayed FGF23 adaptation. In that study, FGF23 was only measured at two time points, preoperatively and on postoperative day 6, which may have missed transient or delayed changes in its regulation. Alternatively, persistent bone turnover or residual metabolic effects of chronic PHPT may have maintained FGF23 production independent of acute calcium changes. These considerations highlight the complex regulation of FGF23, with calcium acting as one of several interacting stimuli rather than as a single regulator.

Although not the primary aim of this study, our findings raise therapeutic considerations. In hypercalcemic conditions such as vitamin D intoxication or PHPT, targeting FGF23 or its downstream pathways may represent a novel adjunct to the standard approach, particularly when conventional therapies, such as hydration, diuretics, bisphosphonates, or calcitonin, are insufficient [21].

Our findings may also have implications for managing postoperative hypocalcemia after total thyroidectomy [22,23]. Although vitamin D supplementation is commonly used preoperatively, its effectiveness remains inconsistent [24,25]. We hypothesize that high preoperative levels of 1,25(OH)_2_D may induce FGF23 expression, which in turn suppresses renal 1α-hydroxylase and activates 24-hydroxylase, leading to insufficient levels of 1,25(OH)_2_D postoperatively. Addressing baseline FGF23 levels may support individualized preoperative management and reduce the risk of hypocalcemia.

Limitations of this study include its cross-sectional design, which precludes causal inferences. The findings are limited to PHPT and Graves’ disease and may not be generalizable to other hypercalcemic conditions. In addition, though we used composite indices to estimate renal hydroxylase activity and PTH efficiency, these surrogate markers are not validated and should be interpreted with caution.

## Conclusions

FGF23 may respond to hypercalcemia in both PHPT and Graves’ disease, possibly acting as a calcium-sensitive hormone. However, its compensatory effects on calcium regulation appear limited in these conditions. Further longitudinal and mechanistic studies are warranted to clarify the regulatory role of FGF23 in calcium metabolism and its broader relevance in endocrine pathophysiology.
